# Adsorption of an anionic dye (Congo red) from aqueous solutions by pine bark

**DOI:** 10.1038/s41598-019-53046-z

**Published:** 2019-11-11

**Authors:** Khaoula Litefti, M. Sonia Freire, Mostafa Stitou, Julia González-Álvarez

**Affiliations:** 1 Department of Chemical Engineering, School of Engineering, Universidade de Santiago de Compostela, Rúa Lope Gómez de Marzoa, s/n, 15782 Santiago de Compostela, Spain; 2 Department of Chemistry, Faculty of Sciences, University Abdelmalek Essaâdi, B.P. 2121, Mhanech II, 93002 Tétouan, Morocco

**Keywords:** Pollution remediation, Chemical engineering

## Abstract

*Pinus pinaster* bark, an abundant by-product from the timber industry, has been studied as a potential low-cost adsorbent for the removal of Congo red (CR) dye from wastewaters. Surface morphological and physico-chemical characteristics of pine bark were analysed using Fourier transform infrared (FTIR) spectroscopy, scanning electron microscopy (SEM), determination of the point of zero charge (pH_PZC_) and elemental analysis. Assays were performed to determine the wavelength for the maximum absorbance and the stability with time of CR solutions depending on concentration and/or pH, which resulted to be a very significant parameter. Adsorption studies were conducted on batch mode to study the effect of contact time (till 7 days), pH (2–9), adsorbent dosage (1–10 g L^−1^) and temperature (25–60 °C). The bark adsorption capacity at equilibrium varied between 0.3 and 1.6 mg g^−1^ and the equilibrium adsorption percentage between 23.4 and 100% depending on adsorbent dosage, temperature and pH at an initial CR concentration of 5 mg L^−1^. Kinetic data for the removal of CR by pine bark were best fitted by the pseudo-second-order kinetic model. The equilibrium data fitted well with the Freundlich model. Thermodynamic analysis indicated that the adsorption process is exothermic and spontaneous.

## Introduction

The textile industries use dyes to colour their products and consume substantial volumes of water. As a result, they generate considerable amounts of coloured wastewaters^1^. It is estimated that more than 100000 commercially available dyes with over 7 × 10^5^ t of dyestuff are produced annually^2,3^ and 5–10% of dyestuffs are lost in the industrial effluents^4,5^. The direct discharge of these effluents into natural streams and rivers causes important environmental problems due to their contribution to high organic loading, toxicity and contamination by colour^6^, reducing light penetration and photosynthesis which affects the aquatic life^7^. In addition, most of dyes are either toxic or mutagenic and carcinogenic^8–10^.

Conventional technologies for removing dyes from industrial effluents include chemical, biological and physicochemical treatments^11,12^. Adsorption on activated carbons is one of the most efficient processes for wastewater treatment^13–15^, however, the current goal is substituting activated carbons for efficient, low-cost and highly available biosorbents. Thus, many studies have been performed to investigate the use of low-cost adsorbents for the removal of dyes from aqueous solutions^16–18^. Agricultural wastes or by-products have been used without or with a light processing, reducing production costs by using renewable, easily available and cheap raw materials and eliminating energy costs associated with thermal treatment. However, other biomass materials have been modified by physical or chemical treatments, such as carbonization or the use of chemical activating agents, to achieve better adsorption characteristics and to make them more effective^16^.

The treatment of dye effluents is difficult due to their different synthetic origins and aromatic structures; moreover, they are not biodegradable. Congo red (CR) is a benzidine-based anionic diazo dye that can cause allergic reactions and can be metabolized to benzidine, a carcinogenic product^19^. Depending on pH, various molecular structures of CR can be present in aqueous solution^20^, which hinders its elimination.

*Pinus pinaster* bark is an abundant by-product from the timber industry in Galicia (NW of Spain), mainly used as fuel or in horticulture. In previous works, the removal of metal cations and phenol from aqueous solutions using *Pinus pinaster* bark has been demonstrated^21,22^. The aim of the present work was to test for the first time, as far as we know, the use of *Pinus pinaster* bark for the removal of CR from aqueous solutions. An effective use of pine bark as biosorbent of CR and other dyes from wastewaters would contribute not only to its valorisation but also to process sustainability. Batch adsorption experiments were carried out to analyse the influence of contact time, pH, adsorbent dosage and temperature on CR adsorption by pine bark. The effect of pH was particularly emphasized and, thus, the stability of the dye solutions at different initial concentration and pH was also studied. In addition, adsorption kinetics and equilibrium were analysed applying mathematical models to experimental data.

## Materials and Methods

### Adsorbent

*Pinus pinaster* bark was supplied by the company Aserpal S.A. (Grupo Losán S.A., Galicia, NW Spain) specialized in the elaboration of fine wood surfaces. It was air-dried to a moisture content close to equilibrium (approximately 20%, on dried basis). It was ground in a hammer mill and the fraction of particles with size between 0.5 and 2 mm was selected. In order to extract soluble compounds that could colour the water and interfere with the analytical determination of the dye, the bark was pre-treated twice with water at 25 °C for 4 h at a solid/liquid ratio of 1/10 (g mL^−1^). The bark, once filtered and air-dried, was stored in plastic bags until their use for analysis and adsorption experiments. It was verified that after the second pre-treatment stage the filtrate came out colourless.

### Chemicals

Congo red (CR) (Direct Red 28, C.I. 22120, azo dye, C_32_H_22_N_6_Na_2_O_6_S_2_, molecular weight 696.7) was selected as anionic dye. The pH was adjusted with 0.1 M NaOH or 0.1 M HCl solutions. All chemicals were of analytical grade. The dye solutions (5–100 mg L^−1^) were prepared by diluting a stock CR solution (100 mg L^−1^) with distilled water.

### Characterization of the adsorbent

The carbon, hydrogen and nitrogen, contents of *Pinus pinaster* bark were determined using an Elemental Combustion System (Thermo Finnigan Flash 1112 model). The O content was calculated by difference. The ash content was determined according to the ASTM D1102-84 norm^23^.

The morphological features and surface characteristics of the adsorbent before and after CR adsorption were obtained by scanning electron microscopy (SEM) using a ZEISS EVO LS 15 microscope (Germany) at 200 magnifications.

FTIR spectra of the adsorbent before and after adsorption were recorded by triplicate with a Varian FT-IR 670 model in the range of 4000–400 cm^−1^. Each sample was finely ground, mixed with KBr at a ratio of 1/50 (mg/mg) and pressed to prepare the pellet.

The pH of the point of zero charge (pH_PZC_) of *Pinus pinaster* bark was determined according to the procedure described by Moreno-Castilla *et al*.^24^.

### Stability of CR dye solutions

In order to determine the wavelength for the value of maximum absorbance (λ_max_) and its change with pH, a CR solution of 5 mg L^−1^ was used and pH was modified from 2 to 12 using 0.1 M HCl or 0.1 M NaOH. Then, the visible absorption spectra of the solutions were recorded at room temperature with a UV–Vis spectrophotometer (V-630 UV/VIS JASCO, Japan).

To study dye stability, experiments were carried out at 25 °C by contacting 100 mL of the dye solution at the selected pH, natural pH (pH = 6) or pH = 2, and at different concentrations (5, 50 or 100 mg L^−1^) in a thermostatic orbital water bath shaker (UNITRONIC-OR SELECTA, Spain) at a shaking rate of 100 rpm. Samples were withdrawn at predetermined times (1, 2, 6 and 7 days) and the absorbance was measured spectrophotometrically at the λ_max_ previously determined. The pH of dye solutions was adjusted by using 0.1 M HCl and 0.1 M NaOH solutions.

### Adsorption kinetic experiments

Batch adsorption experiments were conducted in a thermostatic orbital water bath shaker (UNITRONIC-OR SELECTA, Spain) at a shaking rate of 100 rpm. 100 mL of the dye solution (5 mg L^−1^) were put in contact with the selected amount of adsorbent in stoppered Erlenmeyer flasks and the effect of contact time, solution initial pH (2–9), adsorbent dosage (1, 5 and 10 g L^−1^) and temperature (25, 40 and 60 °C) on the adsorption process was studied using the one-factor-at-a-time method. Solution pH changed during the adsorption process from an initial pH of 2, 6 and 9 to a final pH of 2.2, 4.5 and 4.7, respectively, due to variations of the functional groups in pine bark.

At the selected times, the samples were withdrawn, the suspensions were centrifuged and the residual dye concentration on the supernatant was determined from the calibration curve by measuring the absorbance at the λ_max_ previously determined. Measurements were made in duplicate and the results averaged.

The amount of dye adsorbed onto pine bark at time t, or adsorption capacity, q_t_ (mg g^−1^), was calculated by the following mass balance relationship:

1 $${q}_{t}=\frac{({C}_{0}-{C}_{t})\,V}{m}$$

where C_0_ is the initial dye concentration (mg L^−1^), C_t_ is the dye concentration at any time t, V is the volume of solution (L) and m is the mass of adsorbent (g of oven dried (o.d.) pine bark). The dye removal efficiency was determined as:

2 $$ \% \,{\rm{Adsorption}}=100\,(\frac{{C}_{0}-{C}_{t}}{{C}_{0}})$$

### Adsorption equilibrium experiments

Equilibrium adsorption studies were conducted in a thermostatic orbital water bath shaker (UNITRONIC-OR SELECTA, Spain) at a shaking rate of 100 rpm by contacting at 25 °C and natural pH, 100 mL of the dye solutions of different initial concentrations, from 5 to 100 mg L^−1^, with 1 g of pine bark (on dried basis) for 7 days. This time was considered enough to reach equilibrium.

### Theory

#### Adsorption Kinetics

The Lagergren’s first order kinetic equation was applied in its linear form:

3 $$\log \,({q}_{e}-{q}_{t})=\,\log ({q}_{e})-\frac{{k}_{1}}{2.303}\,t$$

where q_t_ and q_e_ are the amounts of dye adsorbed (mg g^−1^) at t and equilibrium time (h), respectively, and k_1_ represents the first-order rate constant (h^−1^).

The adsorption data were also analysed in terms of the Ho’s pseudo-second-order model^25^ in the following form:

4 $$\frac{t}{{q}_{t}}=\frac{1}{{k}_{2}{q}_{e}^{2}}+\frac{1}{{q}_{e}}t$$

With k_2_ (g mg^−1^ h^−1^), the pseudo-second-order rate constant. The initial sorption rate (h_0_, mg g^−1^ h^−1^), was determined when t approaches to zero, as follows:

5 $${h}_{0}={k}_{2}{q}_{e}^{2}$$

#### Adsorption mechanism

Intra-particle diffusion model based on the model proposed by Weber and Morris^26^ is commonly used for identifying the adsorption mechanism for design purposes. For most adsorption processes, the uptake varies almost proportionately with t^1/2^ rather than with the contact time and can be represented as follows:

6 $${q}_{t}={K}_{id}{t}^{1/2}+I$$

where I is the intercept and K_id_ is the rate constant of intra-particle diffusion (mg g^−1^ h^−1/2^).

#### Thermodynamic study

Thermodynamic parameters such as Gibb’s free energy (ΔG°), enthalpy change (ΔH°) and entropy change (ΔS°) for the adsorption of dye on pine bark have been determined by using the following equations:

7 $$\Delta {G}^{0}=\Delta {H}^{0}-T\Delta {S}^{0}$$

8 $$\mathrm{ln}(\frac{{q}_{e}}{{C}_{e}})=\frac{\Delta {S}^{0}}{R}-\frac{\Delta {H}^{0}}{R}(\frac{1}{T})$$

where C_e_ is the equilibrium concentration (mg L^−1^), T is temperature (K) and R is the ideal gas constant.

#### Adsorption Isotherm

To determine the adsorption isotherms, two commonly used models, the Freundlich and Langmuir equations, were applied to explain the dye-*Pinus pinaster* bark interactions.

The Langmuir isotherm Eq. (9) assumes monolayer adsorption onto a surface containing a finite number of adsorption sites^27^. This isotherm can be represented in a linear form as follows:

9 $$\frac{{C}_{e}}{{q}_{e}}=(\frac{1}{{K}_{a}{q}_{m}})+\frac{{C}_{e}}{{q}_{m}}$$

where q_m_ is the maximum adsorption capacity (mg g^−1^) and *K*_*a*_ is the constant related to the energy of adsorption (L mg^−1^).

The essential features of the Langmuir isotherm may be expressed in terms of equilibrium parameter or separation factor, R_L_, which is a dimensionless constant**:**

10 $${R}_{L}=\frac{1}{1+{K}_{a}{C}_{0}}$$

The value of R_L_ indicates the adsorption nature to be either unfavourable (R_L_ > 1), linear (R_L_ = 1), favourable (0 < R_L_ < 1) or irreversible (R_L_ = 0).

The Freundlich adsorption isotherm^28^, which assumes that adsorption occurs on heterogeneous surfaces, can be expressed linearly as:

11 $${ln}\,{q}_{e}=\,{ln}\,{K}_{f}+\frac{1}{n}\,{ln}\,{C}_{e}$$

where K_f_ and n are the isotherm constants which indicate the adsorption capacity and intensity, respectively.

## Results and Discussion

### Adsorbent characterization

The chemical composition of the adsorbent was analysed, and the results revealed that contains: C, 48.2%, O, 45.1%, H, 6.5% and N, 0.2% (% w/w). As any lignocellulosic material, the main content corresponded to carbon, whereas nitrogen and ash contents (0.45%, w/w) were very low.

**Figure 1:**
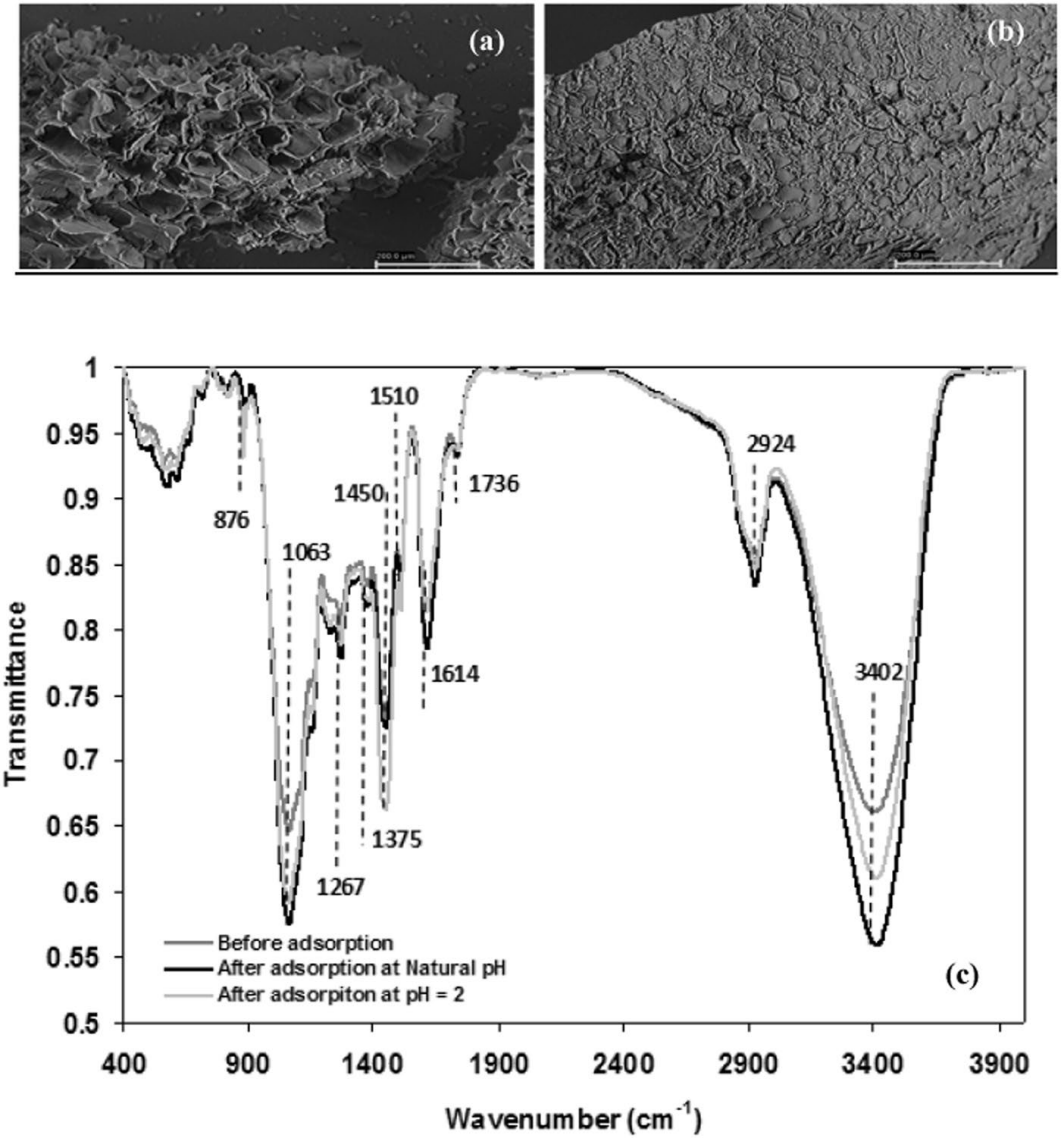
SEM images of *Pinus pinaster* bark (**a)** before and (**b**) after Congo red adsorption and (**c**) FTIR spectra of pine bark before and after Congo red adsorption at pH = 6 (natural) and pH = 2.

SEM analysis is widely used to study the morphological features and surface characteristics of adsorbents. It reveals their surface texture and porosity and plays an important role in determining the surface availability for adsorption^29^. Thus, the SEM micrographs of *Pinus pinaster* bark before and after Congo red adsorption are presented in Fig. 1a,b, respectively. It was observed that the adsorption caused significant changes in the surface texture of the bark. Before adsorption, its surface was heterogeneous, evidencing the presence of pores with different shapes and sizes. However, after dye adsorption a thick layer of dye covering the pores was observed and, as a result, the surface presented a smooth morphology. This is in good agreement with other authors who also found surface morphological changes after dye loading^30^.

*Pinus pinaster* bark, as other agro-industrial materials, contains polar functional groups such as alcohols, aldehydes, ketones, carboxylic, phenolic and others, constituting active sites for the adsorption of dye molecules. To analyse the effect of dye adsorption on these functional groups, FTIR spectra of pine bark before and after adsorption were compared at two of the pH essayed, natural pH (pH = 6) and pH = 2 (Fig. 1c). The peak assignments according to literature^18,31^ are presented in Table 1. As shown in Fig. 1c, the spectra have similar characteristics, even though some peaks are shifted, or their intensities changed. Thus, after CR adsorption, the absorption band at 3402 cm^−1^ shifted to higher wavenumbers and increased in intensity, more significantly at the higher pH. Moreover, the band at 1614 cm^−1^ increased at natural pH, whereas diminished slightly at pH = 2. The spectra before and after dye adsorption also indicate that the band at 1267 cm^−1^ corresponding to –SO_3_ group increased after adsorption. These changes suggested interactions between the functional groups of the bark and dye molecules as reported in literature^18,32^.

**Table 1:** FTIR spectra peaks assignment.

Wavenumber (cm^−1^)			Assignments^a^
Before adsorption	After adsorption		
	Natural pH	pH = 2	
3402	3413	3410	O–H and N–H stretching
2924	2925	2925	Aliphatic C–H stretching
1736	1736	1736	Ketone/aldehyde CO stretching
1614	1616	1612	C=O stretching
1510	1510	1510	Secondary amine groups
1450	1450	1450	Aromatic ring vibration
1375	1375	1383	C-H bending
1317	1317	1315	CH_2_wagging
1267	1267	1267	- SO_3_ stretching
1157	1155	1155	C–O–C asymmetrical stretching
1063	1063	1063	C–O, CC, and C–C–O stretching
876	876	876	Glycosidic linkage

^a^Yaneva and Georgieva^18^.

To understand the adsorption mechanism, it is necessary to determine the concentration of the surface-active groups, i.e. the point of zero charge (pH_PZC_) of the adsorbent. Thus, the pH_PZC_ of the bark was found to be 3.4. This means that the adsorption of cationic forms is favoured at pH > pH_PZC_ as the surface is negatively charged, whereas anion adsorption is favoured at pH < pH_PZC_^32^. Therefore, it is expected that low pH favours the removal of Congo red since it’s an anionic dye.

### Stability of CR dye solutions

The degradation of Congo red and the influence of pH in the UV-Vis absorption, previously reported^33^, have been confirmed.

**Figure 2:**
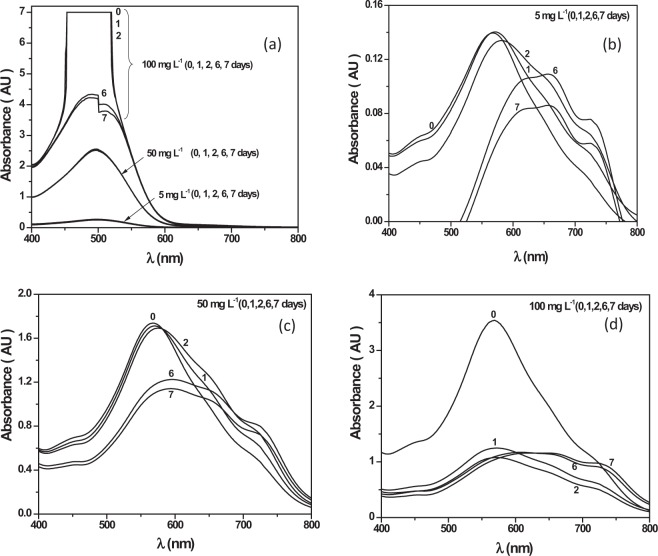
Stability of Congo red solutions with time at different initial concentrations (**a**) natural pH, (**b**) pH = 2 and 5 mg L^−1^, (**c**) pH = 2 and 50 mg L^−1^ and (**d**) pH = 2 and 100 mg L^−1^.

To analyse the colour change and stability with time of aqueous Congo red solutions at various initial concentrations, stability tests at two of the pH essayed (natural and 2) were performed, avoiding the pH range between 2.3 and 4 corresponding to the dye transition zone. As shown in Fig. 2a, the colour is stable for 7 days at the natural pH and low initial concentrations (below 50 mg L^−1^). However, at the highest concentration, the absorbance of the main band of the spectrum decreased markedly with time. At pH 2, a pronounced shift and a significant decrease in the absorbance of the main absorption band were observed (Fig. 2b–d). This behaviour was more evident when the dye concentration increased, and it is probably related to the formation of molecular aggregates of CR^18^. Accordingly, adsorption kinetic experiments were conducted at the lowest initial concentration essayed (5 mg L^−1^).

### Adsorption kinetics experiments

#### Effect of initial solution pH on CR adsorption

**Figure 3:**
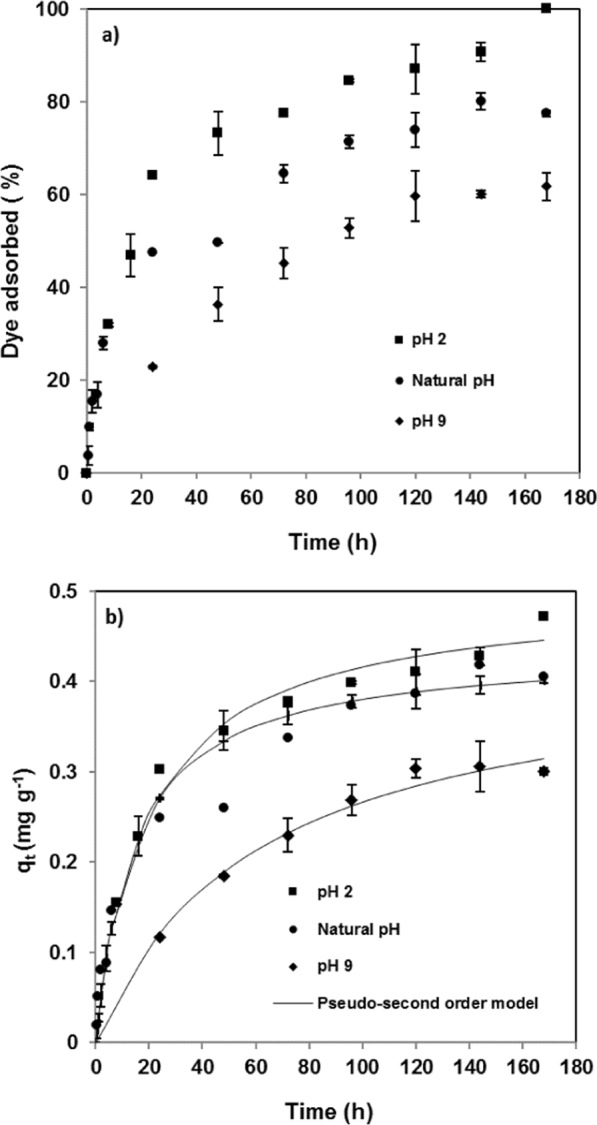
Effect of initial pH on (**a**) removal efficiency and (**b**) adsorption capacity. Experimental data and fitting to pseudo-second order kinetic model (temperature = 25 °C; initial dye concentration = 5 mg/L; adsorbent dose = 10 g L^−1^)

pH affects the adsorption behaviour of the systems essayed, increasing or decreasing the adsorption capacity, because it modifies the ionization state of the binding groups of both adsorbate and adsorbent, as mentioned before. Therefore, it is necessary to determine the best pH for CR adsorption by pine bark. Thus, experiments were performed at pH from 2 to 9, using an initial dye concentration of 5 mg L^−1^, a solid/liquid ratio of 10 g L^−1^ and a temperature of 25 °C. As seen from Fig. 3a the highest adsorption rate was attained at pH = 2, whereas at pH = 9 adsorption was much slower, which implied that at least seven days were necessary for guaranteeing adsorption equilibrium.

According to the point of zero charge of 3.4 found for pine bark, the predominant functional groups are anionic at alkaline pH, whereas at acid pH the charges are positive. Moreover, the isoelectric point of Congo red is 3, and, therefore, its molecular structures are predominantly negative at pH > 3, being in non-dissociated and cationic forms at pH < pH_PZC_. That is, at the two low pH essayed (natural and 2) the participation of electrostatic interactions between the bark surface and Congo red will be negligible, mainly due to the repulsion between similarly charged molecules^18^.

The maximum equilibrium adsorption capacity of 0.47 mg g^−1^ corresponding to almost 100% of removal efficiency was achieved at pH = 2 (Fig. 3a,b). Usually, the adsorption anionic dyes, such as Congo red, increases as the pH decreases. Thus, at the highest pH essayed (pH = 9), both the removal efficiency and the equilibrium adsorption capacity decreased to 61.7% and 0.3 mg g^−1^, respectively. The removal efficiencies at equilibrium were higher than those found for *Pinus radiata* cone biomass of 60.5 and 5.75% at pH of 3.55 and 10.95, respectively, for an initial concentration of 20 mg L^−1^ at 30 °C and an equilibrium time considerably lower, within 100 min^17^. Apart from the negative charge on the bark surface, the OH^−^ ions compete for the adsorption sites^17,18,20^. In acid conditions, probably, the hydrophobic and van der Waals interactions and H-bonding become dominant in the adsorption mechanism^34^.

The experimental data were analysed by the pseudo-first-order and pseudo-second-order kinetic models. As seen in Fig. 3b, the experimental data were well fitted by the pseudo-second-order model (R^2^ > 0.98). The model parameters together with the correlation coefficients are shown in Table 2. As can be proved, the predicted values for q_e_ were close to the experimental ones. On the contrary, the pseudo-first-order model did not apply well for all pH essayed (0.93 > R^2^ > 0.81). This result suggested that the overall rate of CR adsorption could be controlled by chemical processes between CR and *Pinus pinaster* bark^35^.

**Table 2:** Pseudo-second-order and intra-particle diffusion kinetic parameters for the removal of Congo red by pine bark.

Pseudo-second-order model						
			Equilibrium Adsorption	k_2_	q_e_	R^(a)^
			%	(g mg^−1^ h^−1^)	(mg g^−1^)	
pH^(b)^		2	94.4	0.101	0.50	0.992
		6 (Natural)	76.6	0.156	0.44	0.987
		9	61.9	0.038	0.43	0.982
Adsorbent dosage (g L^−1^)^(c)^ pH = 6 (Natural)		1	29.0	0.004	2.51	0.911
		5	66.8	0.015	1.00	0.975
		10	76.6	0.156	0.44	0.987
Adsorbent dosage (g L^−1^)^(c)^ pH = 2		1	24.5	0.004	2.00	0.870
		5	80.0	0.008	1.25	0.955
		10	94.4	0.101	0.50	0.992
Temperature (°C)^(d)^		25	94.5	0.219	0.50	1.000
		40	89.7	0.363	0.46	1.000
		60	79.7	0.605	0.41	1.000
**Intra-particle diffusion model**						
	**First stage**			**Second stage**		
	**K**_**1d**_ **(mg g** ^**−1**^ **h** ^**0.5**^ **)**	**I**_**1**_ **(mg g** ^**−1**^ **)**	**R** ^**(a)**^	**K**_**2d**_ **(mg g** ^**−1**^ **h** ^**0.5**^ **)**	**I**_**2**_ **(mg g** ^**−1**^ **)**	**R** ^**(a)**^
pH = 2	0.0605	−0.006	0.994	0.0198	0.202	0.954
pH = 6 (Natural)	0.0525	−0.003	0.978	0.0243	0.116	0.881
pH = 9	0.0276	−0.006	0.995	—	—	—

^(a)^p < 0.0001.

^(b)^Adsorbent dosage = 10 g L^−1^; Temperature = 25 °C.

^(c)^Temperature = 25 °C.

^(d)^Adsorbent dosage = 10 g L^−1^; Natural pH.

**Figure 4:**
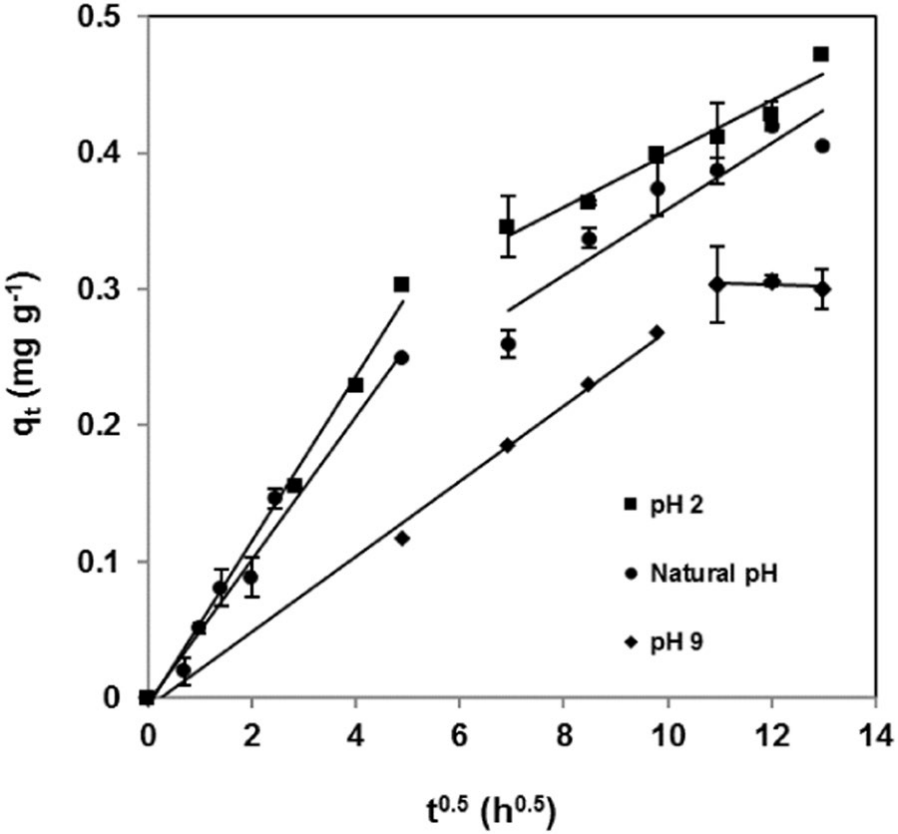
Application of intra-particle diffusion model for Congo red adsorption by *Pinus pinaster* bark (temperature = 25 °C; initial dye concentration = 5 mg L^−1^; adsorbent dose = 10 g L^−1^).

Additionally, to identify the diffusion mechanism, adsorption data were analysed using the intra-particle diffusion model (Eq. 6). As shown in Fig. 4, multilinearity was found for all pHs, which confirmed the occurrence of multiple adsorption stages, external mass transfer followed by intra-particle diffusion. Dye molecules were quickly transported to the bark external surface through film diffusion. After that, dye molecules entered the pores of pine bark by intra-particle diffusion in a slower step, as confirmed by the K_id_ values (Table 2). It can be concluded that intra-particle and external diffusion occurred simultaneously^36^.

#### Effect of the adsorbent dosage

**Figure 5:**
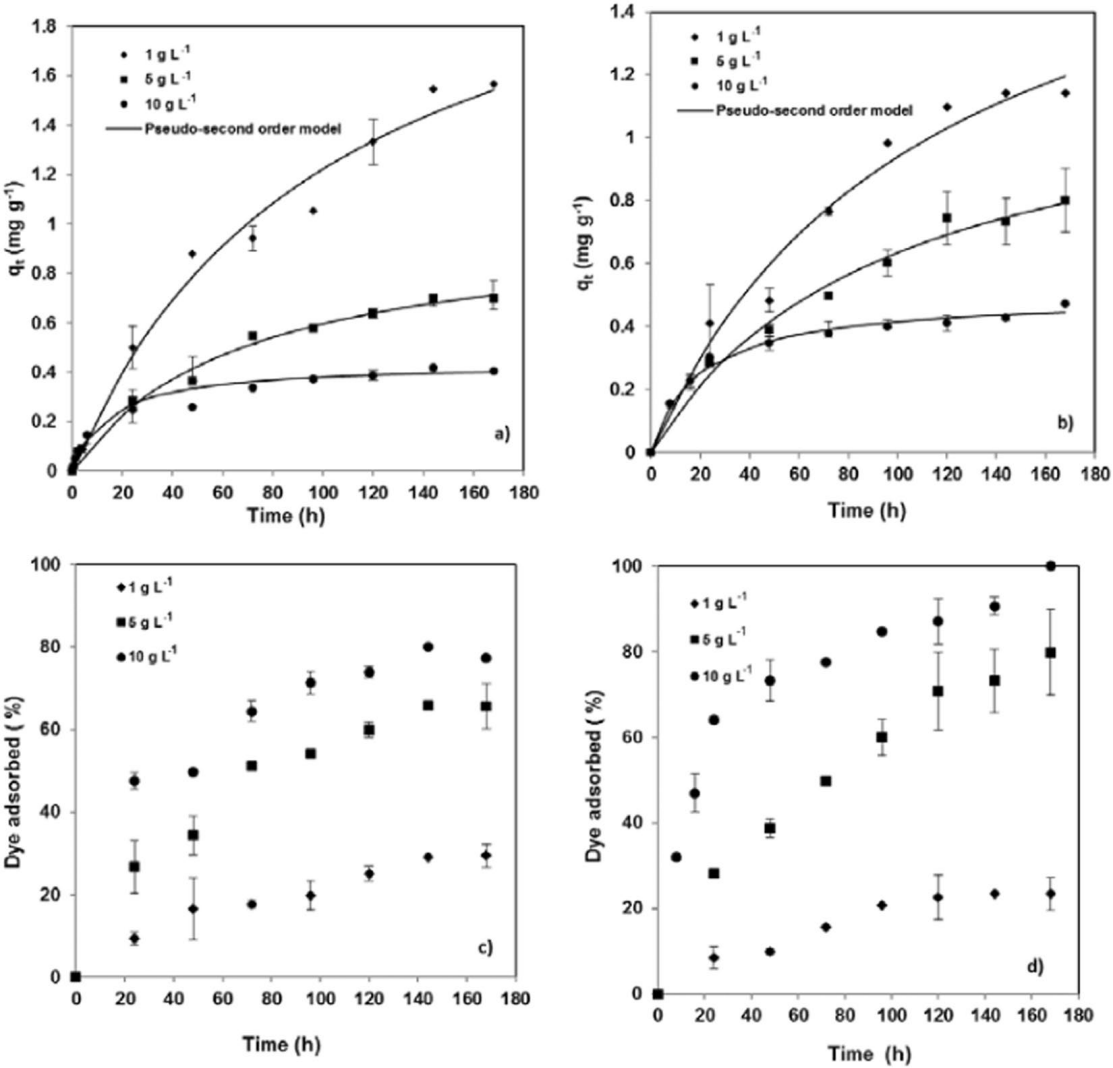
Effect of the pine bark dosage on Congo red adsorption capacity at (**a**) natural pH and (**b**) pH = 2 and on removal efficiency at (**c**) natural pH and (**d**) pH = 2 (temperature = 25 °C; initial dye concentration = 5 mg L^−1^). Experimental data and fitting to pseudo-second order kinetic model.

The effect of pine bark dosage, which was varied from 1 to 10 g L^−1^, on CR adsorption was studied at 25 °C with a Congo red solution of 5 mg L^−1^ at the two pHs leading to the highest dye removal, 2 and natural pH. The kinetic behaviour was also well represented by the pseudo-second-order model (Fig. 5a,b and Table 2), which confirmed that the adsorbent dosage did not influence the adsorption mechanism.

As shown in Fig. 5c,d, increasing pine bark dosage significantly improved CR removal efficiency, from 29.4 to 77.4% at natural pH and from 23.4 to 100% at pH 2. This behaviour can be explained by the greater number of active sites when the amount of adsorbent is increased^16^. On the contrary, it has been found that the adsorption capacity decreased when the adsorbent dosage was increased as shown in Fig. 5a,b for both pHs. Additionally, when decreasing the adsorbent dosage the equilibrium sorption capacity (q_e_) was lower than the expected maximum capacity (Table 2). This behaviour can be explained by the lower dye concentration that hinders adsorbent-adsorbate interactions^37^.

#### Effect of temperature on dye adsorption kinetics

**Figure 6:**
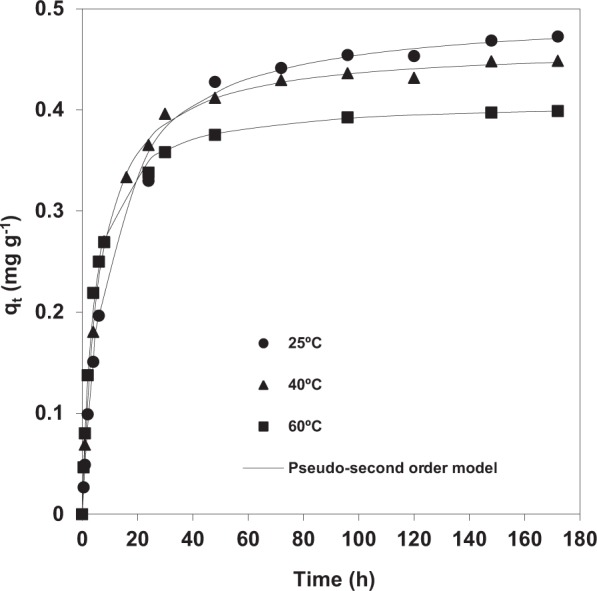
Effect of temperature on Congo red adsorption capacity of *Pinus pinaster* bark at natural pH. Experimental data and fitting to pseudo-second order kinetic model (initial dye concentration = 5 mg L^−1^; adsorbent dose = 10 g L^−1^).

The effect of temperature (in the range 25–60 °C) on CR adsorption by pine bark was studied using a CR solution of 5 mg L^−1^ at the natural pH of the solution and an adsorbent dosage of 10 g L^−1^. Natural pH was selected for studying the influence of temperature as provided a relatively high adsorption rate and more stable solutions than pH = 2. A slight influence of temperature on the removal of CR by *Pinus pinaster* bark was observed (Fig. 6). Thus, the adsorption capacity decreased with increasing temperature from 25 °C to 60 °C, which indicates that the process is exothermic.

#### Thermodynamic studies

The thermodynamic behaviour for the adsorption of CR on pine bark was investigated. Thermodynamic parameters such as the Gibb’s free energy (ΔG°), entropy (ΔS°) and enthalpy (ΔH°) changes were calculated by Eqs (7) and (8) and their values are presented in Table 3. The negative value of ΔG° at 25 °C indicated the feasibility and spontaneity of CR adsorption on pine bark, being less favoured at higher temperatures. Additionally, the value of ΔH° < 84 kJ/mol confirmed that the adsorption was physical^18^. As a result, the weakening of hydrogen bonds and van der Waals interactions at higher temperatures resulted in the weakening of physical interactions between the active sites of pine bark and the dye, decreasing the removal efficiency^38^. The negative value of ΔS° suggested a decrease in randomness at the solid/liquid interface, and that no significant changes occurred in the internal structure of pine bark during CR adsorption. The negative value of ΔH° indicated the exothermic nature of adsorption, as mentioned above.

**Table 3:** Langmuir and Freundlich isotherm parameters and thermodynamic parameters for the removal of Congo red by pine bark.

Isotherm models (25 °C, Natural pH and 10 g L^−1^)					
q_m_ (mg g^−1^)	K_a_ (L mg^−1^)	R^2^	K_F_	n	R^2^
Langmuir isotherm			Freundlich isotherm		
3.92	0.26	0.98	0.96	2.77	0.99
**Thermodynamic parameters (C**_**0**_ ** = 5 mg L** ^− **1**^ **, Natural pH and 10 g L** ^− **1**^ **)**					
**Temperature (K)**	**ΔG° (kJ mol** ^**−1**^ **)**	**ΔH° (kJ mol** ^**−1**^ **)**		**ΔS° (kJ mol** ^**−1**^ **)**	
298	−1.47				
313	0.15	−33.62		−0.11	
333	2.30				

Arrhenius equation rearranged to a linear relationship between the rate constant and temperature, as shown by Eq. 12, was applied to determine the activation energy of the adsorption process (*E*_*a*_):

12 $$\mathrm{ln}\,{k}_{2}=\,\mathrm{ln}\,A-\frac{{E}_{a}}{RT}$$

where A is the Arrhenius factor, R is the ideal gas constant and T is temperature (K). The value of the activation energy, 23.9 kJ mol^−1^, lower than 40 kJ mol^−1^, confirms the physisorption of CR on pine bark^39^.

#### Adsorption equilibrium isotherm

In this study, two of the most widely used isotherms, Freundlich and Langmuir models, were used for describing experimental equilibrium data for Congo red adsorption on pine bark at 25 °C, natural pH and adsorbent dosage of 10 g L^−1^. Both isotherms (Eqs 9 and 11) fitted well the data in terms of correlation coefficients, although the Freundlich model gave a slightly better fit (Table 3), indicating that adsorption occurs on a heterogeneous surface. The value of n > 1 indicates that the adsorption is favourable at 25 °C. The R_L_ value (Eq. 10), which was determined using the parameters of the Langmuir isotherm for the initial concentration of 5 mg L^−1^, is between 0 and 1 (0.43), therefore, *Pinus pinaster* bark is confirmed as a favourable adsorbent for Congo red dye.

## Conclusion

Congo red adsorption by pine bark depended strongly on pH. pH affected not only the surface charges on the adsorbent but also the stability of Congo red solutions related with the formation of aggregates of the dye molecules. This was more evident when pH was decreased and when initial dye concentration was increased. Apart from pH, adsorption percentage and capacity were influenced by adsorbent dosage and temperature. Natural pH provided the highest adsorption rate, stable dye solutions and satisfactory adsorption yields. The adsorption of Congo red by pine bark was demonstrated by the morphological changes in the bark surface and the changes in intensities and shifts of some functional groups in the FTIR spectra. It was found that Congo red adsorption by pine bark followed the pseudo-second-order kinetic model and that intra-particle and external diffusion occurred simultaneously. Freundlich isotherm provided the best fit to the experimental data showing a heterogeneous and multi-layer adsorption. Finally, thermodynamic analysis indicated that Congo red adsorption was a spontaneous, exothermic and a physical process in nature. This work demonstrated the capacity of water-treated pine bark for the removal of Congo red from aqueous solutions. However, although high adsorption percentages were achieved, the time needed to reach equilibrium was too long compared to other biomass adsorbents. Therefore, alternative pretreatments for pine bark must be tested in order to increase adsorption rate and efficiency.
